# Rapid Simultaneous Determination of Telmisartan, Amlodipine Besylate and Hydrochlorothiazide in a Combined Poly Pill Dosage Form by Stability-Indicating Ultra Performance Liquid Chromatography

**DOI:** 10.3797/scipharm.1006-10

**Published:** 2011-01-04

**Authors:** Santaji Nalwade, Vangala Ranga Reddy, Dantu Durga Rao, Inabathina Koteswara Rao

**Affiliations:** 1 Analytical Research and Development, Integrated Product Development, Dr. Reddy’s Laboratories Ltd., Bachupally, Hyderabad-500 072, India; 2 Department of Chemistry, J.N.T. University, Kukatpally, Hyderabad-500 072, A.P., India

**Keywords:** Validation, Stability, UPLC, Simultaneous, Degradation, Method development

## Abstract

A simple, precise and rapid stability-indicating ultra-performance liquid chromatography (UPLC) method is developed for the simultaneous quantitative determination of Telmisartan, Amlodipine besylate and Hydrochlorothiazide from their innovative poly pill combination drug product in the presence of degradation products. It involves a 100 mm x 2.1 mm, 1.7 μm C-18 column. The separation is achieved on a simple gradient method. The mobile phase A contains a mixture of sodium perchlorate buffer pH 3.2 (0.053M): acetonitrile in the ratio 90:10, *v/v*, and mobile B contains a mixture of sodium perchlorate buffer pH 3.2 (0.053M): acetonitrile in the ratio 20:80, *v/v*. The flow rate is 0.6 mL min^−1^ and the column temperature is maintained at 55°C.The gradient program (T/%B) is set as 0/5, 1.2/5, 1.6/40, 4/40, 4.1/5 and 4.5/5. The detector wavelength is 271 nm for Hydrochlorothiazide and Telmisartan and 237 nm for Amlodipine. The retention times of Telmisartan, Amlodipine, and Hydrochlorothiazide are 3.6 minutes, 3.2 minutes and 0.9 minutes; respectively. The total runtime for the separation of the three active compounds and their degradation products is 4.5 minutes. The described method is validated with respect to system suitability, specificity, linearity, precision and accuracy. The precision of the assay method is evaluated by carrying out six independent assays of T, A and H (0.032 mg mL^−1^ of T, 0.004 mg mL^−1^ of A, 0.01 mg mL^−1^ of H). The accuracy of the method is evaluated in triplicate at three concentration levels, i.e. 50%, 100% and 150% of target test concentration (0.64 mg mL^−1^ of T, 0.08 mg mL^−1^ of A, 0.2 mg mL^−1^ of H). The described method is linear over the range, 16 to 48 μg mL^−1^ for T, 2 to 6 μg mL^−1^A and 5 to 15 μg mL^−1^ for H. The method is fast and suitable for high-throughput analysis allowing the analysis of about 250 samples per working day.

## Introduction

Cardiovascular diseases (CVDs) are the disorders of heart and blood vessels and primarily include coronary heart disease, hypertension, cerebrovascular disease, peripheral artery disease, rheumatic heart disease, congenital heart disease and heart failure. CVDs are the major cause of death in developed countries and also are rapidly emerging as a main cause of death in the developing world. An estimated 17.5 million people died from CVDs till 2005, representing almost 30% of all the global deaths. It is projected that almost 20 million people will die from CVDs by 2015. The major risk factors involved in CVDs are high low density lipoprotein (LDL) cholesterol, raised blood pressure, increased serum homocysteine level and platelet aggregation, which are primarily caused by unhealthy diet, physical inactivity and tobacco use. A novel polypill formulation is developed using drugs Telmisartan, Amlodipine besylate and Hydrochlorothiazide for CVDs.

**Fig. 1.**
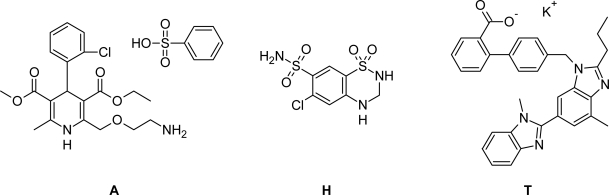
Structure of Amlodipine besylate (A), Hydrochlorothiazide (H) and Telmisartan (T)

Telmisartan (T) is an angiotensin II receptor (type AT_1_) antagonist used in the management of hypertension. Telmisartan prevents the constriction (narrowing) of blood vessels (veins and arteries). Telmisartan is a non-peptide molecule and chemically described as potassium 4′-[(1,7′-dimethyl-2′-propyl-1*H*,3′*H*-2,5′-bibenzimidazol-3′-yl)-methyl]biphenyl-2-carboxylate (Fig. 1).

Hydrochlorothiazide (H) is a thiazide diuretic (water pill) that decreases the amount of fluid in the body by increasing the amount of salt and water lost in the urine. Hydrochlorothiazide is used to lower blood pressure and to decrease edema (swelling), it is chemically described as 6-chloro-3,4-dihydro-2*H*-1,2,4-benzothiadiazine-7-sulfonamide 1,1-dioxide (Fig. 1).

Amlodipine besylate (A) is in a class of drugs called beta-blockers. Beta-blockers affect the heart and circulatory system (arteries and veins). Amlodipine besylate is used to lower blood pressure, lower heart rate, reduce chest pain (angina), and to reduce the risk of recurrent heart attacks. It is chemically described as 3-ethyl 5-methyl 2-[(2-amino-ethoxy)methyl]-4-(2-chlorophenyl)-6-methyl-1,4-dihydropyridine-3,5-dicarboxylate benzenesulfonate (Fig. 1).

The ever-increasing need for speed and efficient use of time in the pharmaceutical and other fields, there is demand for the development of fast and high throughput analytical procedures. The rapid quantitative determination of combination drugs with big difference in label claims (40 mg for T and 5 mg for A) with shorter run times is a challenge. For UPLC based assays, the processes of reducing analysis time while adequately resolving analytes from degradation products is often accomplished with column with small particles. The theoretical advantages for small particles are to get well resolved peaks with high theoretical plates over small concentration.

Present drug stability test guidance Q1A (R2) issued by international conference on harmonization (ICH) [1] suggest that stress studies should be carried out on a drug product to establish its inherent stability characteristics, leading to identification of degradation products and hence supporting the suitability of the proposed analytical procedures. It also requires that analytical test procedures for stability samples should be stability indicating and they should be fully validated.

Accordingly, the aim of the present study was to establish inherent stability of Telmisartan, Amlodipine besylate, and Hydrochlorothiazide through stress studies under a variety of ICH recommended test conditions [1–3] and to develop a rapid stability-indicating reverse phase assay method [4–6].

Literature survey reveals that a variety of spectrophotometric and chromatographic methods including UV, colorimetric determination, ratio derivative, and a stability-indicating HPLC methods have been reported for determination T A and H either single or in combination with other drugs [7–12]. Whereas no liquid chromatography method has been reported for simultaneous quantitative determination of T, A and H in the combined dosage form.

Hence a rapid simple reproducible Ultra performance liquid chromatography method was developed for simultaneous quantitative determination of T, A and H in polypill pharmaceutical dosage forms in the presence of degradation products.

## Experimental

### Chemicals and reagents

Standards and tablets (40 mg of Telmisartan, 5 mg of Amlodipine besylate, 12.5 mg of Hydrochlorothiazide) were supplied by Dr. Reddy’s laboratories limited, Hyderabad, India. The HPLC grade acetonitrile and methanol, analytical grade sodium per chlorate and disodium hydrogen phosphate were purchased from Merck, Darmstadt, Germany. Water was prepared by using Millipore MilliQ Plus water purification system

### Instrumentation

Acquity UPLC™ system (Waters, Milford, USA) used consisting of a binary solvent manager, a sample manager and a UV detector. The out put signal was monitored and processed using empower software, water bath equipped with MV controller (Julabo, Seelbach, Germany) was used for hydrolysis studies. Photo stability studies were carried out in a photo stability chamber (Sanyo, Leicestershire, UK). Thermal stability studies were performed in a dry air oven (MACK Pharmatech, Hyderabad, India).

### Chromatographic conditions

The chromatographic column used was Acquity UPLC BEH shield RP-18, 100 mm x 2.1 mm i.d with 1.7 μm particles. The mobile phase A contains a mixture of sodium perchlorate buffer pH 3.2(0.053M): acetonitrile (90:10, *v/v*) and mobile phase B contains a mixture of sodium perchlorate buffer pH 3.2(0.053M): acetonitrile (20:80, *v/v*). The flow rate was 0.6 mL min^−1^ and column temperature was maintained at 55°C. The detection wavelength was 271 nm for Hydrochlorothiazide and Telmisartan, and 237 nm for Amlodipine besylate. The diluent contains a mixture of sodium perchlorate buffer pH 3.2(0.053M): methanol in the ratio 90:10, *v/v.*

### Preparation of standard solutions

Stock standard solutions of T, A and H (0.54 mg mL^−1^ of T, 0.2 mg mL^−1^ of A, 0.5 mg mL^−1^ of H) were prepared by dissolving an appropriate amounts of the compounds in methanol. Working solutions 0.032 mg mL^−1^ of T, 0.004 mg mL^−1^ of A, 0.01 mg mL^−1^ of H were prepared from above stock solution in mobile phase for assay determination.

### Preparation of sample solution

Weighed and crushed twenty tablets into a clean and dry mortar-pestle. An equivalent to 20 mg of H (64 mg of Telmisartan, 8 mg of Amlodipine besylate) was transferred to a 100 mL volumetric flask, added 15 mL of 0.1% orthophosphoric acid and kept on rotary shaker for 20 minutes. Added 50 mL of methanol and sonicated for 20 minutes. Made the volume to 100 mL with methanol (0.64 mg mL^−1^ of T, 0.08 mg mL^−1^ of A, 0.2 mg mL^−1^ of H). About 5 mL of supernant solution was taken and diluted to 100 mL with diluent to get working concentrators (0.032 mg mL^−1^ of T, 0.004 mg mL^−1^ of A, 0.01 mg mL^−1^ of H). The solution was then filtered through 0.45 μ (Nylon 66- membrane) filter.

## Results and Discussion

### Chromatographic Method Development

The method was optimized to separate major degradation products formed under different stress conditions. The main target of the chromatographic method is to get the separation for closely eluting degradation products, mainly the degradation product at 1.26 RRT (with respect to A) that is eluting between A and T. The degradation samples were run using different stationary phases like C18, C8, and Mobile phases containing buffers like phosphate and perchlorate with different pH (2.5–7) and using organic modifiers like acetonitrile and methanol in the mobile phase. The isocratic method was not working since telmisartan and amlodipine peaks were not separated with a proper resolution and also the degradents of hydrochlorothiazide were not separating from three actives. Hence the method is optimized with a gradient program. As the concentration of amlodipine is very less as compared to telmisartan, so that to improve response, resolution and peak shapes the method was tried at different column temperatures. But the separation, response of peaks and peak shapes were satisfactory in the adopted chromatographic conditions only. It indicated that the gradient method with 10% acetonitrile as organic modifier in mobile phase A and 80% in mobile phase B was successful in separating drugs and all chromatographic degradation products. Some of the trials were summarized as below.

### Selection of Diluent (Extracting solvent):

As the tablets are film coated,and all the active drugs are stable in acidic conditions, the 0.1% orthophosphoric acid is added in order to open the film coating and to use the same assay method for quantification of uniformity of dosage units by UPLC.The trials were 0.1 N HCl also but the peak shapes are not good in 0.1N HCl. The chromatograph shown in Fig.2a. As all the three actives T, A, & H are freely soluble in methanol, so it is used as the extracting solvent.

### Selection of Stationary Phase:

The stationary phases like C8 and C18 were tried but on C8 the peak shape for T and the peak symmetry factor for hydrochlorothiazide is more than 1.7, the separation between A and T is not adequate as shown in Fig.2b. On the other hand the peak shapes for all the three components are good and all the three peaks are well separated from each other and their degradents. So that the column with C18 stationary phase is selected.

### Selection of mobile phase buffer:

The buffers like Phosphate buffer With Buffer with pH 3.2, pH 7.5 and the perchlorate buffer with pH 2.5 and 3.2 were tried as mobile phase buffer. But with pH 2.5, pH 7.5 phosphate and per chlorate buffer with pH 2.5 the results were not enough good in terms of peak separation and peak shapes, shown in Fig. 2c. On the other hand the perchlorate buffer with pH 3.2, the results were found satisfactory so that the mobile phase with pH 3.2 perchlorate buffer is finalized.

### Optimization of gradient program:

Before going to gradient program, an isocratic program with organic modifier methanol and acetonitrile were tried but all the three components are eluted at the same retention time with both methanol and acetonitrile. As we are using the column temperature 55°C the acetonitrile is preferred as an organic modifier rather than methanol. The different gradient programs tried are shown in the Fig. 2d.

### Finalization of Chromatographic conditions

**Fig. 3.**
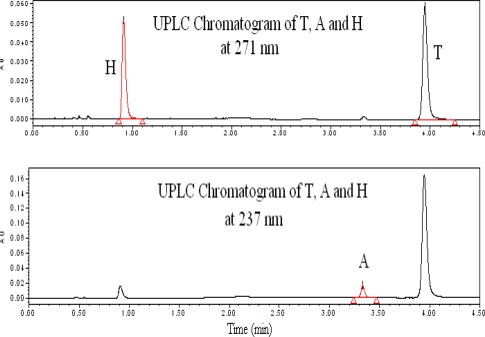
Typical chromatogram of Telmisartan, Amlodipine besylate and Hydrochlorothiazide at 271 nm and 237 nm from drug product.

By considering all the experiments the chromatographic conditions are finalized. The chromatograph with finalized chromatographic condition was shown in the Fig.3.

Further the analysis was performed for pharmaceutical dosage forms of both the label claim tablets (40 mg T, 5 mg A, 12.5 mg H and 20 mg T, 2.5 mg A, 6.25 mg H). The assay (% of active drug content present in the tablets with respect to its label claims) results for drug product, 99.8%, 99.5%,of T, 99.7%, 98.8% of A and 100.1%, 99.8% of H, respectively.

Analysis also performed (n = 3) separately for Telmisartan, Telmisartan and Hydrochlorothiazide tablets and Amlodipine besylate and Hydrochlorothiazide tablets. The assay results for Telmisartan were 99.5%, 99.7%, 99.9%. The assay results for Telmisartan and Hydrochlorothiazide were, 99.7%, 99.5% and 99.9% for Telmisartan and 99.3%, 98.5% and 99.1% for Hydrochlorothiazide. The assay results for Amlodipine besylate and Hydrochlorothiazide were, 99.5%, 99.7% and 98.7% for Amlodipine besylate and 99.8%, 98.5% and 99.3% for Hydrochlorothiazide. It indicated that, adopted UPLC method also can be used separately for assay estimation of Telmisartan tablets, simultaneous estimation of Telmisartan and Hydrochlorothiazide tablets and simultaneous estimation of Amlodipine besylate and Hydrochlorothiazide tablet.

## Method validation

### System suitability

In order to find the adequate peak separation (resolution) and repeatability of the proposed method, suitability parameters including retention factor, selectivity and asymmetry factor were investigated and the results were abridged in Table 1.

### Specificity

The specificity of an analytical method is the ability of the method to determine the analyte response in the presence of additional components such as impurities, degradation products and matrix [4]. The solution of analytical placebo (containing all the excipients except T, A, & H) was prepared according to the sample preparation procedure and injected. To identify the interference by these excipients, a mixture of inactive ingredients, standard solutions, and the commercial pharmaceutical preparations including T, A, and H were analyzed by the developed method.

The specificity of the method was also evaluated to ensure there were no interference products resulting from forced degradation.

### Placebo Interference

**Fig. 4.**
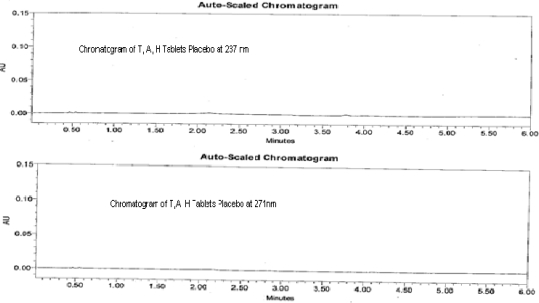
Typical chromatogram of Telmisartan, Amlodipine besylate and Hydrochlorothiazide tablets placebo blend at 271 nm and 237 nm from drug product

The placebo (excepients present in the tablet) sample were prepared as per the test method and analyzed in the UPLC. The Fig.4. shows there is no peaks at the retention time of the T, A, & H in the chromatograph indicates that there is no placebo interference.

### Forced degradation studies

Stress testing of a drug substance can help to identify the likely degradation products, which can help to establish the degradation pathways and the intrinsic stability of the molecule.

All stress decomposition studies were performed at an initial drug concentration 0.64 mg mL^−1^ of T, 0.08 mg mL^−1^ of A, 0.2 mg mL^−1^ of H. The degradation conditions are selected on the basis of literature survey [13–17].

### Water induced degradation

Water hydrolysis was performed in purified at 60 °C for 6 hours. Fig. 5a shows the major degradation found at RRT 0.83 with respect to H. and all the major and minor degradation products were well separated from T, A, and H peaks. The peak purity is checked for T, A, and H and the results are summarized in Table 2.

### Hydrogen peroxide induced degradation

For hydrogen peroxide-induced degradation, the studies were carried out at room temperature in 1% hydrogen peroxide for 6 hours. The Fig.5a. shows the major degradation found at RRT 0.841 with respect to H and at RRT 1.056 and 1.272 with respect to A. All the major and minor degradation products were well separated from T, A, and H peaks. The peak purity is checked for T, A, and H and the results are summarized in Table 2.

### Acid induced degradation

Acid hydrolysis was performed in 1N HCl at 60 °C for 6 hours. Fig. 5a shows the major degradation found at RRT 0.842 with respect to H. and all the major and minor degradation products were well separated from T, A, and H peaks. The peak purity is checked for T, A, and H and the results are summarized in Table 2.

### Base induced degradation

The study in basic solution was carried out in 0.1N NaOH at 60 °C for 6 hours. The Fig. 5b. shows all the three T, A, and H were stable in 0.1N NaOH and all the minor degradation products were well separated from T, A, and H peaks. The peak purity is checked for T, A, and H and the results are summarized in Table 2

### Heat induced degradation

The drug product was exposed to dry heat at 105 °C for 24 hours. Samples were withdrawn at appropriate time and subjected to UPLC analysis after suitable dilution (0.032 mg mL^−1^ of T, 0.004 mg mL^−1^ of A, 0.01 mg mL^−1^ of H).The Fig. 5b. shows that the drug product containing T, A, and H is thermally stable. The peak purity is checked for T, A, and H and the results are summarized in Table 2.

### Photo-degradation

Photo degradation studies were carried out at according to option 2 of Q1B in ICH guidelines [3]. Samples were exposed to light for an overall illumination of 1.2 million lux hours and an integrated near ultraviolet energy of 200 watt hm^2^. Samples were withdrawn at appropriate time and subjected to UPLC analysis after suitable dilution (0.032 mg mL^−1^ of T, 0.004 mg mL^−1^ of A, 0.01 mg mL^−1^ of H). The drugs T, A and H are stable under photolytic condition. The peak purity is checked for T, A, and H and the results are summarized in Table 2.

### Linearity

Linearity solutions were prepared from stock solution at six concentration levels from 50 to 150% of analyte concentrations (16 to 48 μg mL^−1^ for T, 2 to 6 μg mL^−1^A and 5 to 15 μg mL^−1^ for H). The linear regression analysis of T, A, and H were constructed by plotting the peak area of the analytes (y) versus analytes concentration in (x) axis. The calibration curves were linear in the range of 16 to 48 μg mL^−1^ for T, 2 to 6 μg mL^−1^A and 5 to 15 μg mL^−1^ for H with a correlation coefficient of more than 0.999 for all the three drugs. The slope, Y-intercept and correlation coefficient were calculated and summarized in Table 3.

### Precision

The precision of the assay method was evaluated by carrying out six independent assays of T, A and H (0.032 mg mL^−1^ of T, 0.004 mg mL^−1^ of A, 0.01 mg mL^−1^ of H) test samples against qualified reference standard. The percentage of RSD of six assay values was calculated. Different analyst from the same laboratory evaluated the intermediate precision of the method. The R.S.D. values of intra- and inter-day studies for T, A, and H confirming good precision of the method (Table 4).

### Accuracy

The accuracy of an analytical method expresses the nearness between the reference value and found value. The accuracy of the method was evaluated in triplicate at three concentration levels, i.e. 50%, 100% and 150% of target test concentration (0.64 mg mL^−1^ of T, 0.08 mg mL^−1^ of A, 0.2 mg mL^−1^ of H) in tablets. The results obtained are shown in Table 5.

### Solution Stability and Mobile Phase Stability

The solution stability of T, A and H was carried out by leaving the test solution in tightly capped volumetric flask at room temperature for 48 hrs. The same sample solution was assayed for a 24 hours interval up to the study period against freshly prepared standard solution of T, A and H. The mobile phase stability was also carried out by assaying the freshly prepared standard solution for 24 hours interval up to 48 hours. The mobile phase preparation was kept constant during the study period. The percentage RSD of assay of T, A and H was calculated for the study period during mobile phase and solution stability experiments. The % RSD of the assay of T, A, and H during solution stability and mobile phase experiments were within 1% and it indicates that both standard and test preparation and mobile phase were stable for 2 days on bench top at room temperature.

## Conclusions

The established UPLC method proves to be simple, linear, precise, accurate and specific. The total runtime was 4.5 minutes within which three drugs and their degradation products were separated. The method was validated and shows satisfactory data for all the method validation parameters tested. The Developed method is stability indicating and can be used for simultaneous quantitative determination of the drugs T, A and H in presence of degradation products in stability by the industry. The adopted UPLC method also can be used separately for assay estimation of Telmisartan tablets, simultaneous estimation of Telmisartan and Hydrochlorothiazide tablets and simultaneous estimation of Amlodipine besylate and Hydrochlorothiazide tablets.

## Figures and Tables

**Fig. 2a.**
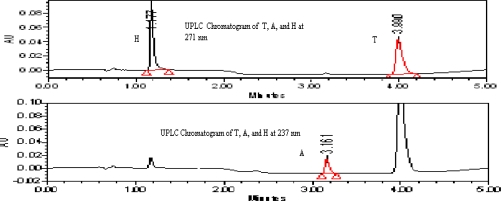
Chromatogram of T, A and H at 271 nm and 237 nm from drug product, as 0.1 N HCl used in the sample preparation instead of 0.1 % OPA

**Fig. 2b.**
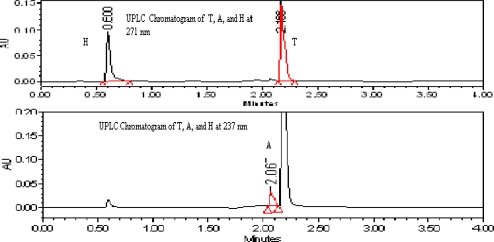
Chromatogram of T, A and H at 271 nm and 237 nm from drug product by using 100 mm x 2.1 mm, 1.7 μm C-8 column with gradient program as 0/5, 1.0/5, 1.2/40, 3/40, 3.3/5 and 4.0/5.

**Fig. 2c.**
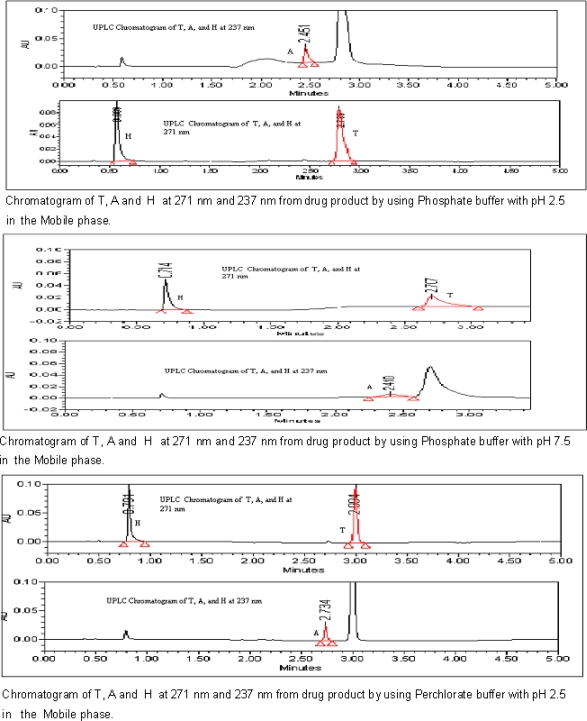
Chromatograms of T, A, and H from drug product by using different mobile phase buffers.

**Fig. 2d.**
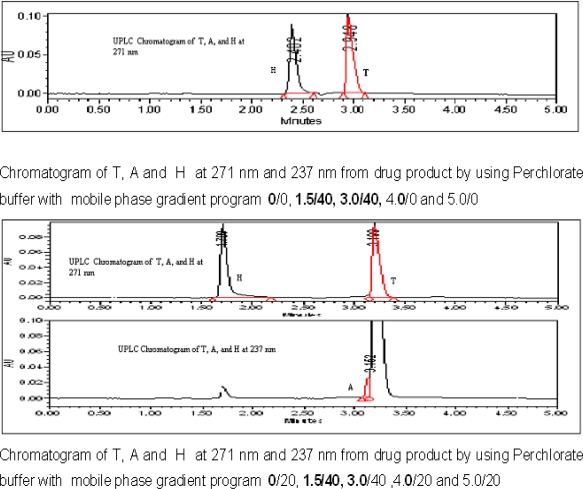
Chromatograms of T, A, and H from drug product by using different gradient programs.

**Fig. 5a.**
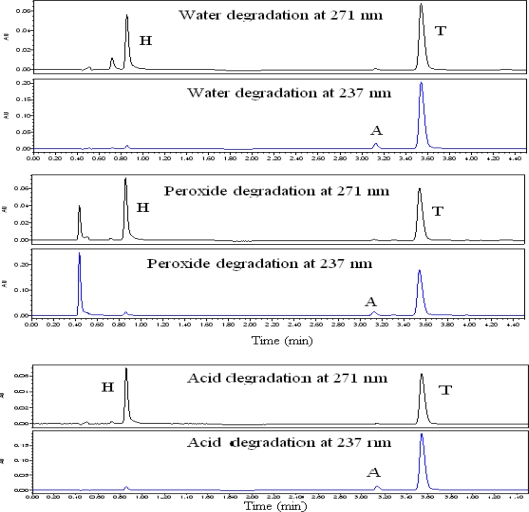
Typical chromatograms of Telmisartan, Amlodipine besylate and Hydrochlorothiazide at 271 nm and 237 nm for force degradation study

**Fig. 5b.**
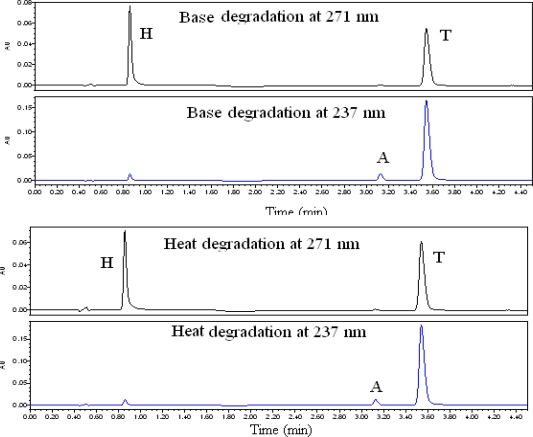
Typical chromatograms of Telmisartan, Amlodipine besylate and Hydrochlorothiazide at 271 nm and 237 nm for force degradation study

**Tab.1.** System suitability parameters for T, A and H

**System suitability test parameters**	**T**	**A**	**H**
Retention time(min) (mean ± S.D., n = 5)	3.944 ± 0.0018	3.332 ± 0.0010	0.913 ± 0.001
Repeatability of retention time; R.S.D. % (n = 5)	0.0460	0.0300	0.3342
Repeatability of peak area; R.S.D.% =(S.D./mean) x 100	0.1501	0.5403	0.0752
Resolution (Rs)	8.3	36.9	–
Tailing factor (asymmetric factor)	1.2	1.2	1.4
Retention factor(k’)	2.944	2.332	0.082
USP plate count	34765	43314	2974

**Tab. 2.** Peak purity results of T, A and H

**Stress Condition**	**Purity Angle**			**Purity Threshold**			**Purity Flag**		
	**T**	**A**	**H**	**T**	**A**	**H**	**T**	**A**	**H**

Heat Stress	0.070	1.118	0.114	0.538	4.580	1.140	No	No	No
Aqueous Stress	0.057	0.902	0.131	0.499	3.973	1.384	No	No	No
Acid Stress	0.064	1.108	0.108	0.493	4.301	1.125	No	No	No
Base Stress	0.065	1.306	0.122	0.536	5.057	1.212	No	No	No
Peroxide Stress	0.060	1.727	0.241	0.533	5.776	1.202	No	No	No

**Tab. 3.** Linearity

**Analyte**	**Concentration range**	**r^a^**	**Slope**	**intercept**	**95% confidence interval for intercept**	**Potential assay bias**
T	16.024–48.073 μg.ml^−1^	0.9999	6282.0	−888.486	877.9552 & −2656.9267	−0.4
A	2.020–6.020 μg.ml^−1^	0.9996	10044.1	−14.5193	596.3377 & −625.3762	0.0
H	5.000–15.000 μg.ml^−1^	0.9998	13499.7	−827.248	2710.7834 & −4365.2787	−0.6

a Correlation coefficient.

**Tab. 4.** Intra-day and inter-day Precision results of T, A and H from tablets (n = 6)

	**Active Name**	**Pre-1**	**Pre-2**	**Pre-3**	**Pre-4**	**Pre-5**	**Pre-6**	**% RSD**	**% Mean**

		**% Assay**							
Intra-day precision	T	100.2	99.7	100.2	100.4	99.4	100.6	0.45	100.1
	A	99.0	99.3	98.6	99.4	101.2	99.2	0.90	99.5
	H	98.9	98.8	99.3	99.9	100.1	99.8	0.54	99.5

Inter-day precision	T	98.0	100.0	100.9	99.0	99.3	100.4	1.04	99.6
	A	99.9	99.4	98.4	99.4	99.8	99.4	0.53	99.4
	H	100.0	100.6	100.6	99.7	100.0	99.3	0.51	100.0

**Tab. 5.** Accuracy Results of T, A and H from tablets (n = 6)

**Analyte**	**Recovery level**	**Actual Conc. (μg.ml^−1^)**	**Found Conc. (μg.ml^−1^)**	**% Recovery**	**% R.S.D.**	**% Error^a^**
T	50%	320	321.86±0.567	100.58	0.564	0.581
	100%	640	640.51±0.449	100.08	0.449	0.080
	150%	960	964.51±0.638	100.47	0.635	0.470

A	50%	40	39.99±0.480	99.97	0.480	−0.030
	100%	80	79.56±0.903	99.45	0.908	−0.550
	150%	120	119.78±0.194	99.82	0.194	−0.183

	50%	100	993.2±0.492	99.32	0.495	−0.683
H	100%	200	198.94±0.547	99.47	0.549	−0.530
	150%	300	298.50±0.297	99.50	0.298	−0.750

a [found conc. − actual conc./actual conc.] x 100.
